# Evaluation of Different Combinations of Ornamental Perennials for Sustainable Management in Urban Greening

**DOI:** 10.3390/plants12183293

**Published:** 2023-09-18

**Authors:** Enrico Pomatto, Federica Larcher, Matteo Caser, Walter Gaino, Marco Devecchi

**Affiliations:** Department of Agricultural, Forest and Food Sciences, University of Turin, Largo Paolo Braccini 2, 10095 Grugliasco, Italy; enrico.pomatto@unito.it (E.P.); matteo.caser@unito.it (M.C.); walter.gaino@unito.it (W.G.); marco.devecchi@unito.it (M.D.)

**Keywords:** urban horticulture, greening design, resilience, flowerbeds, ground cover, growing performance, floriculture

## Abstract

Ornamental perennial plants play a strategic role in reducing green areas’ management costs, keeping the ground, sparing water, and avoiding weeds. The aim of this research is to evaluate the growing performances of seven combinations of six different ornamental perennial herbaceous species and their role in weed containment under low-maintenance conditions. The experiment was performed for three years (2019–2021) in an open field. The selected species were *Hemerocallis* “Stella de Oro” (A), *Phedimus spurius* (M.Bieb.) “t Hart ‘John Creech” (B), *Tulbaghia violacea* Harv. (C), *Phlox subulata* L. “Trot Pink” (D), *Potentilla neumanniana* Rchb. (E), and *Gaillardia* “Kobold” (F). Four replicates for each combination were tested (28 plots, 4 m^2^ each): AB, CD, EF, AB + CD, AB + EF, CD + EF; AB + CD + EF. No watering or fertilization was performed during the cultivation period. Each year, from April to November, three manual weeding activities were performed and the dry weights of the weeds’ aerial parts were measured. The ground cover performance was evaluated through digital image analysis using the mobile device application Canopeo. Dry aerial perennial biomass variations between the end and beginning of the experiment were calculated. As a result, CD showed the best performance for weed containment (0.5 g m^−2^ weed dry weight in the third year), ground cover (63.1% and 64.3% of plot coverages during the second and third years, respectively), and producing ornamental biomass (4316.8 g m^−2^). The highest total dry amount of harvested weeds was shown by AB + CD + EF (1114.6 g m^−2^), demonstrating that combinations with a higher number of species were less efficient in avoiding weeds. The research allowed us to identify the best combinations to always keep the soil covered and to improve the ornamental and environmental values of urban green spaces under low-maintenance regimes.

## 1. Introduction

Urban green areas are ever-increasingly associated by the scientific community to a series of benefits for the environment and the well-being of humans [1]. It is recognized as having an important role in increasing a city’s resilience and adaptation to climate change effects, thanks to the effect of reducing urban heat islands [2,3]. Furthermore, the proper design and management of urban green areas are key factors for achieving the ONU Sustainable Development Goal n11 “Sustainable Cities and Communities” [4]. Urban green areas provide several ecosystem services that, in many contexts, have to be improved further [5]. Iojă et al. [6] highlighted the necessity of improving urban green connectivity and multifunctionality using all the available spaces to useful convert into green areas, such as, for example, open spaces in schools. Duan et al. [7] observed the positive effects of urban green infrastructures on human health (e.g., physical health, anxiety, and dysphoria) in China. Similarly, Larcher et al. [8] reported that the social-distancing period, imposed by the outbreak of COVID-19 in 2020, increased the need for Italian citizens to have green areas closer to their homes. These results are in line with the study of Ugolini et al. [9], who highlighted that future urban planning needs to consider the proximity of urban green areas accessible to all residents.

In this context, a novel approach to the design of more sustainable and green cities is a priority, considering the use of trees, shrubs, and herbaceous plants. Herbaceous plants are recognized in Northern Europe as having an important role in making cities more resilient (e.g., to climate change) [10]. Sikorski et al. [11] reported that all of the available surfaces in Warsaw, Poland, can be grassed in order to make the city greener. The sustainable approach also requires the reduction in management and the environmental costs of green areas in urban contexts. Silvennoinen et al. [12] estimated the monetary value of urban green areas in Finland, explaining that they can save 90,000 to 270,000 EUR/ha for urban runoff management. Nat et al. [13] highlighted the need to introduce new design systems in the urban parks of Sheffield (UK), suggesting the consideration of lower maintenance costs and the use of more naturalistic planting in the designs. From this perspective, the use of spontaneous vegetation in urban green areas can be a challenge [14]. Indeed, the scientific community recognizes the importance of spontaneous native species to increase the biodiversity in urban contexts [15,16].

At present, the potential of wildflowers and perennial meadows is also recognized by many authors [17,18,19,20]. Mody et al. [21] reported that they allowed a reduction in the management costs of green areas and that this had positive environmental and biodiversity effects. According to Bretzel at al. [22], the use of wildflowers—that also comprise perennial species—in urban degraded areas increases the biodiversity and the awareness of citizens about environmental issues. Other participatory studies highlight that wildflowers meadows are generally appreciated by urban residents who recognize their important aesthetic and environmental values [23,24,25]. Similarly, Lindemann-Matthies and Brieger [26] reported that among the preferred solutions, in addition to the wildflowers meadows, flowerbeds could increase the aesthetic appeal of urban green spaces. Furthermore, flowerbeds can be planted on small surfaces (e.g., roundabouts or green roadsides), where the use of wildflowers is less recommendable. In these conditions, Colombo et al. report the risk that wildflowers are considered by citizens as untidy and uninviting solutions, especially when the life cycle of non-perennial plants ends [27]. In small areas, flowerbeds with ornamental and native perennials allow the maintenance of ground cover, and their diversity in form and color attract pollinators [28]. By covering the ground during the whole year, ornamental perennials obstruct weed growth, thus reducing management costs [29]. Furthermore, Krzyzak et al. [30] considered nature-based solutions for climate adaptation in urban contexts, selecting species for their adaptability to environmental stresses. Many studies focus on different weed-control methods in urban landscape planting beds, including the use of mulching or herbicides [31,32,33]. Other studies explore the potential of bioherbicides for weed control in urban areas [34,35]. However, the potential of ornamental perennials is underexplored. Foo et al. [36] evaluated the efficiency of weed control for twelve ground cover ornamental perennial species separately in New Zealand. As an example, they observed the good performances of *Acaena inermis* Hook.f. “Purpurea” and *Muehlenbeckia axillaris* (Hook.f.) Walp. in covering the ground rapidly, remaining dense throughout the year, and having a good effect on weed control. However, the effect of combinations of ornamental perennials on weed containment is lacking, as well as long-period experiments.

In this context, the aim of this three-year-long research is to evaluate the growing performances of seven combinations of six different ornamental perennial herbaceous species and their role in weed containment under low-maintenance conditions. The experiment is based on the agronomic performances of the selected species and combinations considering spontaneous vegetation as a weed independently by the species it belongs to.

## 2. Materials and Methods

### 2.1. Plant Material and Experimental Design

**Figure 1:**
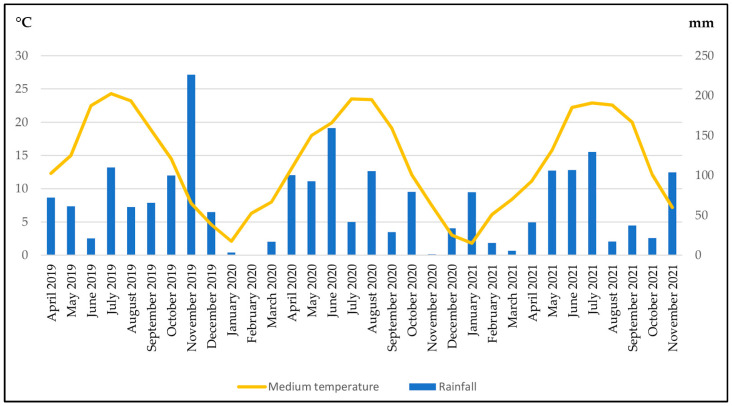
The rainfall and medium temperature detected during the entire experiment (elaboration from ARPA Piemonte data, Bauducchi meteorological station [37]).

The experiment lasted for three years (2019–2021) at the “Vivaio Purpurea di Alberto Peyron” nursery (Piobesi Torinese, Piedmont, Northwest Italy; 44°56′17.96″ N Lat., 7°35′29.46″ E Long.; 238 m a.s.l.). Figure 1 shows the rainfall (mm) and medium temperature (°C) values detected during the entire experiment.

**Figure 2:**
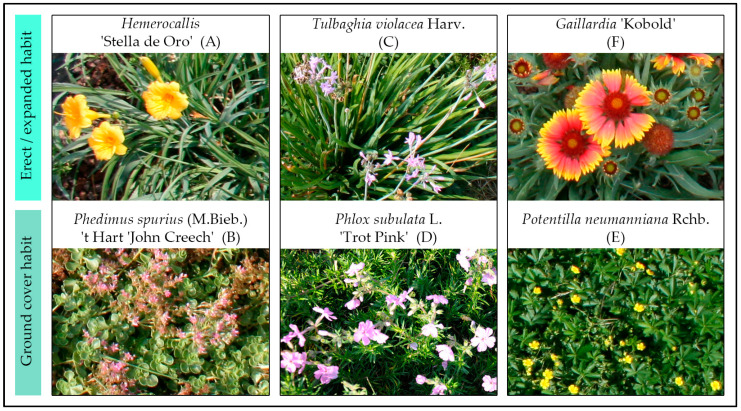
The six species selected for the research.

As shown in Figure 2, six species of ornamental perennial herbaceous species characterized by different growth habits were selected for the research: *Hemerocallis* “Stella de Oro” (A), *Phedimus spurius* (M.Bieb.) ‘t Hart ‘John Creech’ (B), *Tulbaghia violacea* Harv. (C), *Phlox subulata* L. “Trot Pink” (D), *Potentilla neumanniana* Rchb. (E), and *Gaillardia* “Kobol” (F). Table 1 reports the life forms, habits, and blooming times of these species. All of them were selected for their rusticity, good adaptation to urban sun exposure, and low water and nutrient needs.

These species were combined in fixed pairs, composed by one species with a ground cover habit and one with an erect and expanded habit. The three fixed pairs (AB, CD, and EF) were tested alone, combined in three groups of four species (AB + CD, AB + EF, CD + EF), and all together (AB + CD + EF). Overall, seven different combinations were compared.

**Figure 3:**
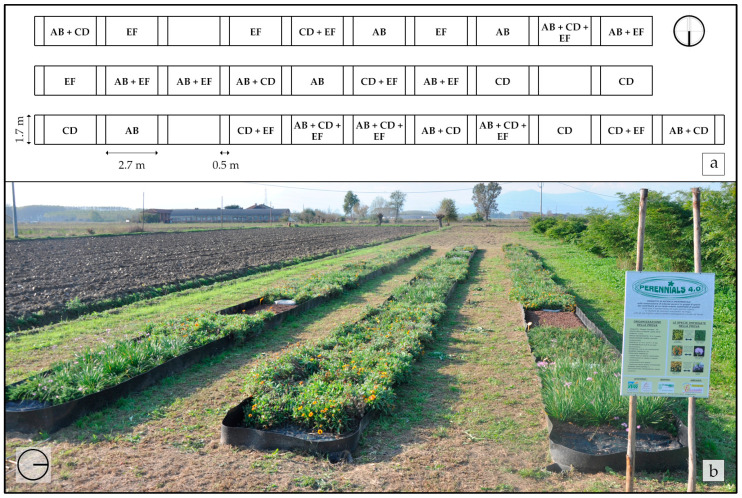
(**a**) The organization of the experimental field with 28 randomized plots of 4 m^2^ with the combinations of the 6 selected ornamental perennials: (*Hemerocallis* “Stella de Oro” (A), *Phedimus spurius* (M.Bieb.) “t Hart ‘John Creech” (B), *Tulbaghia violacea* Harv. (C), *Phlox subulata* L. “Trot Pink” (D), *Potentilla neumanniana* Rchb. (E), and *Gaillardia* “Kobold” (F)). (**b**) The experimental field located in “Vivaio Purpurea di Alberto Peyron” nursery (Piobesi Torinese, Piedmont, Northwest Italy) during autumn in the first year.

The selected combinations of ornamental perennials were randomized and planted in the experimental field exposed to full sun. As shown in Figure 3a, the experimental field was divided into 28 plots, 4 m^2^ each (2.7 × 1.5 m), with a space of 0.5 m between 1 them. Four replicates for each combination were evaluated. In each plot, 15 plants per m^−2^ (i.e., 60 plants per plot^−1^) were planted. These plants were previously grown at “Vivaio Purpurea di Alberto Peyron” (pot diameter: 9 cm). Specifically, 30 plants for each species were used in plots with combinations of 2 species, 15 plants per species in plots with combinations of 4 different species, and 10 plants per species in plots with combinations of all the 6 species. Furthermore, we decided to leave a randomized plot without ornamental plants for each row in order to perform qualitative observations on spontaneous weed development. In these plots, no quantitative data were collected. Figure 3b shows the experimental field during autumn in the first year.

The land preparation for the experimental field started on 25 March 2019. As shown in Figure 3b, the plots are delimited with a high-density polyethylene sheath (METZO^®^PLAST HDPE/T) underground for 0.25 m and above ground for 0.25 m. For the preparation of the substrate, agrarian soil was added with a 10 kg plot^−1^ of coconut fiber (Mattoni Natural Cocco mod. BK5.OW.TOP.MIX5, AGEON S.R.L., Borgo San Dalmazzo, Italy). Furthermore, on 28 March 2019, the plots were fertilized with 0.13 kg plot^−1^ of Labin (mineral organic fertilizer, NPK: 12–12–15, Productos Agricolas MACASA s.l., Igualada, Spain) and 0.12 kg plot^−1^ of Nitrophoska Gold (slow-release fertilizer, NPK: 15–9–16, COMPO EXPERT Italia Srl, Cesano Maderno, Italy). Subsequently, the soil was tilled on 28 March and 5 April 2019. On 19 April 2019, we further fertilized the plots with 0.36 kg plot^−1^ of Labin and 0.16 kg plot^−1^ of Nitrophoska Gold (Compo Agricoltura, Cesano Maderno, MI, Italy), and we weeded the plots in order to avoid competition between the weeds and ornamental perennials in phase engraftment phase, using 0.16 kg plot^−1^ of PotClean 2G (DIACHEM S.P.A., Caravaggio, Italy). On 19 April 2019, the perennial plants were planted within the plots. The plots were then mulched with 148.5 kg plot^−1^ of volcanic lapillus, with an 8–15 mm diameter (Centro Evergreen Turco di Amerio Eugenio & C. SAS, Moncalieri, Italy). An additional 16.5 kg plot^−1^ of volcanic lapillus was distributed to the plots during the first growing season. Finally, on 21 May 2019, manual weeding was performed and the experiment was begun.

During the three years of the experiment, the plots were managed similar to a standard public space: a low-maintenance regime, without irrigation and fertilization, and with manual weeding spread over time. During winter, the plots were cleaned by the dry parts of the perennial plants.

### 2.2. Data Collection

The weed development in each plot and the treatment were calculated. Three manual weeding schedules per year were performed, as shown in Table 2.

The aerial part of the weeds was collected and dried in a forced-draft oven to a constant weight at 90 °C in order to obtain the dry weight. In order to avoid the “edge effect”, we constructed a rectangular bamboo guide measuring 2.3 × 1 m, and we placed it in the middle section of the plots. For the analyses, we considered only the portions of the plots included in this guide. However, all of the surfaces of the plots were cleaned by the weeds. Only those included in the guide were analyzed.

**Figure 4:**
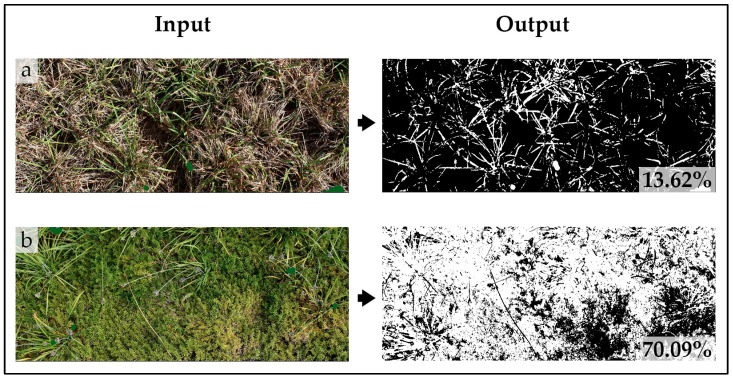
The input and output of the mobile device application Canopeo to obtain the percentages of ground cover by perennials after manual weeding. As an example, the figures show (**a**) the AB (*Hemerocallis* “Stella de Oro” + *Phedimus spurius* (M.Bieb.) “t Hart ‘John Creech”) and (**b**) CD (*Tulbaghia violacea* Harv. + *Phlox subulata* L. “Trot Pink”) combinations at the end of the manual weeding performed in summer in the third year.

Furthermore, after cleaning the plots, one photo per plot was taken perpendicularly at a height of 2 m using a Nikon camera (model D5600, AF-P DX NIKKOR 18–55 mm f/3.5–5.6 G VR). The photos were used, according to Xiong et al. (2019) [41], to evaluate the ground cover percentage through a digital image analysis using the mobile device application Canopeo (Oklahoma State University). The images were firstly processed with Photoshop CC 2017 (Adobe Systems, San Jose, CA, USA) by clipping the selected photos on the rectangular bamboo guide cited above and covering the flowers with green. Indeed, the Canopeo app only recognized the green pixels as representing the canopy cover, returned a binary image composed of white (selection criteria of green canopy satisfied) and black (selection criteria of green canopy not satisfied) pixels, and indicated the percentage of the canopy cover [42]. This made it evident that, in the case of ornamental perennials, if the images were not corrected covering with green the flowers, their covering surface would be underestimated. After the elaborations, all the images were transferred onto an iPhone 6s (Apple, Inc., Cupertino, CA, USA) and processed with the Canopeo app. The vegetation type was set as the cover crop, while the other default values, including the adjustment of the vegetation filter (0.95), were used. Therefore, the ground cover percentages for all the perennials after the manual weeding were obtained. Figure 4 shows two examples of the elaborations performed on Canopeo to obtain these percentages.

Finally, at the beginning and end of the experiment, the dry aerial biomass produced by the ornamental perennials in each plot was measured, after drying them in a forced-draft oven to a constant weight at 90 °C.

### 2.3. Data Analysis

An arcsin transformation was performed on all percentage incidence data before the statistical analysis in order to improve the homogeneity of the variance (Levene test; *p* < 0.05). All the analyzed data were checked for the normality of variance by using the Shapiro–Wilk test (*p* > 0.05). A two-way ANOVA was then performed to examine the influence of years (1, 2, and 3), seasons (spring, summer, and autumn), and their reciprocal interactions on the mean percentages (%) of ground cover. Moreover, regarding all the other traits, mean comparisons were computed using one-way ANOVA. Means were separated according to the Ryan–Einot–Gabriel–Welsch F post hoc test (REGWF) (*p* < 0.05). For all the analyzed parameters not respecting the ANOVA assumptions, the mean differences among species were computed using the non-parametric Kruskal–Wallis test (*p* < 0.05) by a stepwise comparison. All statistical analyses were performed by SPSS software (version 26.0, SPSS Inc., Chicago, IL, USA).

## 3. Results and Discussion

### 3.1. Weed Dry Biomass

An of innovation of our research was the evaluation of the role in weed containment of ornamental perennial species in combination over three years. The mean dry weed biomass (g m^−2^) harvested each year is reported in Table 3. During the first year, no differences were highlighted among the plant combinations, ranging from 9.2 (EF) to 54.9 (AB) g m^−2^. In the second and third years, the CD combination affected weed presence by reducing the weed dry biomass to a very low content (1.4 and 0.5 g m^−2^ in years 2 and 3, respectively) in comparison to CD + EF, AB + EF and AB + CD + EF, and in the third year also to EF. The total dry matter of harvested weeds at the end of the experiment was more than three-times lower in CD (340.6 g m^−2^) in comparison to AB + CD + EF (1114.6 g m^−2^). Indeed, this last combination, including all of the species tested, showed the worst performance for weed containment during the three years, while the combinations with four species showed an intermediate efficiency. This could have been due to the competition among plants of different species or to their different forms that did not allow us to efficiently cover the ground. Eom et al. [43] observed that the most efficient ground cover for avoiding weeds was that characterized by dense foliage, which reduced the light transmittance through the soil and emerged early in spring. Furthermore, among the fifteen herbaceous perennials that they evaluated separately, *Phlox subulata* L., in addition to *Alchemilla mollis* (Buser) Rothm., *Nepeta x faassenii* Bergmans ex Stearn, and *Solidago sphacelata* Raf., showed the best performances for avoiding weeds. This is in line with our findings that, as reported above, recognize *Phlox subulata* L. “Trot Pink” in combination with *Tulbaghia violacea* Harv. (CD) as having the best performance.

The reported results show the better performances of some combinations rather than others for one year or overall during the three years. Instead, the influence of perennial combinations on the weed species grown on the plots was not observed. Indeed, the weed species detected were typical of the site where the experiment was conducted and, independently by the ornamental combinations, in all plots were mainly: *Taraxacum officinale* Weber, *Echinochloa crus-galli* (L.) *p*. Beauv., *Holcus lanatus* L., *Poligonum aviculare* L., *Potentilla indica* (Andrews) Th. Wolf, *Oxalis* spp., *Epilobium* spp., and *Cuscuta* spp.

### 3.2. Percentages of Ground Cover by Perennials

The growing performances of seven combinations of six different ornamental perennial herbaceous species were analyzed, with particular attention being paid to their abilities to cover the ground and avoid weeds. According to Toscano et al. [44], we tested different ornamental perennial species to find those most suitable for urban green areas. Indeed, ornamental ground cover species can be an effective weed deterrent when fully grown, as their dense foliage and growth prevent new weed seedlings from establishing. However, once established, they can vary quite considerably during the years and seasons in their ability to prevent weeds from growing. We selected them for their rusticity and different growth habits (erect/expanded or ground cover).

The effects of the different years (1, 2, and 3) and seasons (spring, summer, and autumn) on the mean percentages (%) of ground cover are reported in Table 4. A general reduction was observed over time, with the highest value in year 1 (66.0%) and the lowest in year 3 (52.8%). Similar behavior was observed during seasons, with the highest mean percentages of ground cover in spring (76.5%) and the lowest in autumn (42.2%). Significant interactions among the effects of the years and seasons were observed.

**Figure 5:**
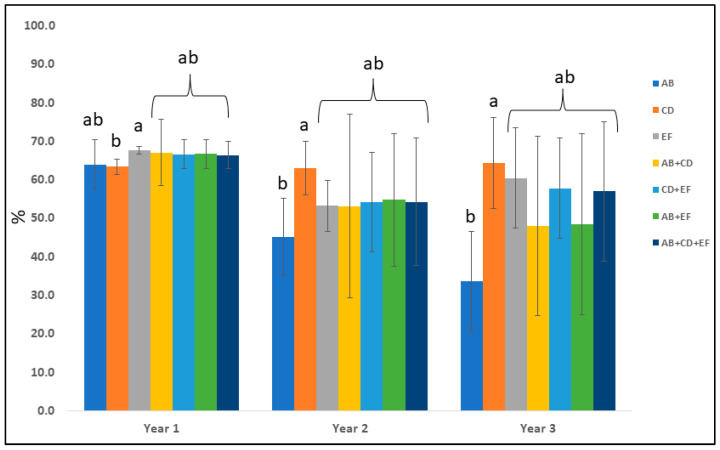
The mean percentages of ground cover by seven perennial plant combinations: AB, CD, EF, AB + CD, CD + EF, AB + EF, and AB + CD + EF (*Hemerocallis* “Stella de Oro” (A), *Phedimus spurius* (M.Bieb.) “t Hart ‘John Creech” (B), *Tulbaghia violacea* Harv. (C), *Phlox subulata* L. “Trot Pink” (D), *Potentilla neumanniana* Rchb. (E), and *Gaillardia* “Kobold” (F)) during years 1, 2, and 3. Means sharing the same letter are not significantly different at *p* ≤ 0.05.

Thus, in Figure 5, the mean percentages of ground cover by means of each perennial combination, during the three years of experimentation, are reported. The results show that all of the combinations tested cover the ground rapidly during the first year of the experiment. Overall, during the first year, the highest value was observed for EF (67.8%) (*Potentilla neumanniana* Rchb. *+ Gaillardia* “Kobold”), which was significantly higher than CD (63.4%) (*Tulbaghia violacea* Harv. and *Phlox subulata* L. “Trot Pink”). Conversely, from the second year onward, this last combination presented the greatest coverage (63.1% and 64.3% for the second and third years, respectively), resulting in being the only combination able to maintain similar mean values during the entire trial period. The lowest percentage was presented by the AB combination (*Hemerocallis* “Stella de Oro” and *Phedimus spurius* (M. Bieb.) “t Hart ‘John Creech”) (45.2% and 33.6% in the second and third years, respectively), with a reduction of −30.3% of coverage from the start.

**Figure 6:**
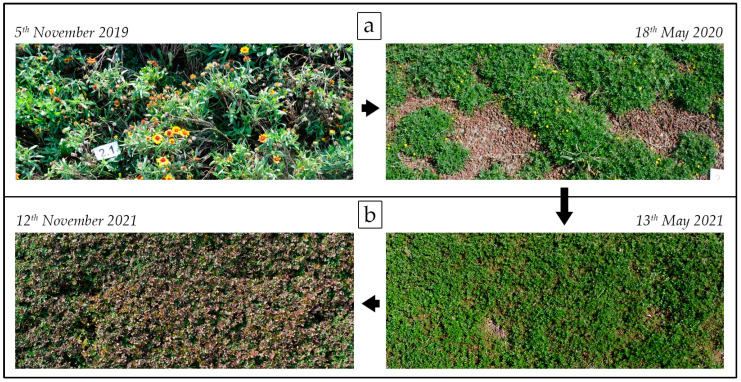
The growth performance of EF (*Potentilla neumanniana* Rchb. + *Gaillardia* “Kobold”): (**a**) from the final manual weeding in year 1 to the first in year 2, the *Gaillardia* “Kobold” plants were almost all dried up and *Potentilla neumanniana* Rchb. alone had not covered the ground well. (**b**) From the second and during the third years, *Potentilla neumanniana* Rchb left alone showed a good performance for ground cover.

The fact that EF was the combination characterized by the best covering effect during the first year, but also by the higher number of weeds in years 2 and 3, as previously discussed, was clearly due to the almost completely dried *Gaillardia* “Kobold” from the first to second years (Figure 6a). Therefore, the good performances of EF during the first year was mainly related to *Gaillardia* “Kobold”, rather than its combination with *Potentilla neumanniana* Rchb. The vigor that characterizes *Gaillardia* “Kobold” plants is well known in the literature. As an example, Süle et al. [45] observed how in Hungary this species becomes potentially invasive when it escapes from gardens. However, we observed that its ability to cover the ground rapidly is limited to the first year after planting, and it is lost in the following years. Additionally, the site conditions and management regime, without irrigation, could have affected this aspect. Indeed, Zollinger et al. [46] report that *Gaillardia* “Kobold” is not recommended for low-water-use landscapes for its tendency to wilt when water is limited; although, it is visually acceptable in a dry landscape setting where rooting volume is not constrained. By contrast, *Potentilla neumanniana* Rchb, existing alone in the EF plot, showed a good performance in ground cover in the second and third years (Figure 6b). This evidence is in line with Martinetti et al. [47], who observed the good water stress tolerance of *Potentilla neumanniana* Rchb on green roofs with very low irrigation levels. Furthermore, in the same conditions in Northern Italy, Tosca et al. [48] reported the good performances of this species in controlling weed invasion due to its good surface cover and allelopathic properties.

**Figure 7:**
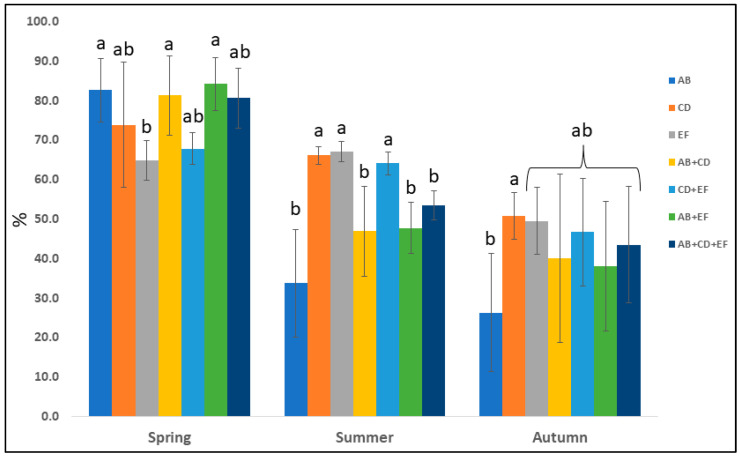
The mean percentages of ground cover by seven perennial plant combinations: AB, CD, EF, AB + CD, CD + EF, AB + EF, and AB + CD + EF (*Hemerocallis* “Stella de Oro” (A), *Phedimus spurius* (M. Bieb.) “t Hart ‘John Creech” (B), *Tulbaghia violacea* Harv. (C), *Phlox subulata* L. “Trot Pink” (D), *Potentilla neumanniana* Rchb. (E), and *Gaillardia* “Kobold” (F)) during spring, summer, and autumn. Means sharing the same letter are not significantly different at *p* ≤ 0.05.

The mean effect of each plant combination in spring, summer, and autumn is reported in Figure 7. On average, during the entire experiment, spring was the season with the highest percentage of ground cover (76.5%), with AB (82.7%), AB + CD (81.3%), and AB + EF (84.2%) combinations being the best ones. Conversely, in summer, these final combinations were the worst, including AB + CD + EF, resulting in mean values significantly lower than CD, EF and CD + EF (66.2%, 67.1% and 64.1%, respectively). Furthermore, in summer, all of the combinations, including AB, showed the worst performances. As can be expected, in autumn, all the perennial combinations resulted in less than 50% (AB showed the lowest value with 26.3%) of ground cover, with the exception of CD (50.8%).

In the Supplementary Materials, Figures S1–S6 show the evolution over time of the different combinations tested. Regardless of the year and season, in Table 5, the detailed data obtained for each time point are reported. During the first year, a general increase in the ground coverage by all the tested combinations was observed, with the exception of EF and AB + EF. In line with the data reported above, during spring, especially in years 2 and 3, the best percentages were observed, with ground coverage values higher than 90% for AB, CD, and AB + CD (96.0%, 94.3%, and 96.7% in year 2, respectively) and AB, AB + CD, AB + EF, and AB + CD + EF (92.0%, 94.6%, 93.6%, and 91.0% in year 3, respectively). Except for the first year, autumn was the season with the lowest values, particularly for AB (2.1% and 0.5% in years 2 and 3, respectively). As previously reported in Figure 1, during the three years of the experiment, the medium temperatures were typical of the site where the experiment was conducted. A temperature pick was registered in November in the first year, followed by a dry period during the following months. The management of the plots without irrigation and the performances of the perennial species described above prove their potential for use in urban green areas. However, these conditions without irrigation and during seasons characterized by low precipitation and high temperatures are also limiting for weeds. So, it is possible that, for weed containment, in addition to the role of the perennial species tested, seasonal conditions also play a role. Probably, if the plots were irrigated or the rainfall was more abundant, the weeds would have grown more. However, this trend probably would have also been observed for the ornamental perennials. Moreover, since the aim of the research was to identify solutions for urban contexts, where conditions are, in general, limited, this aspect can be waived.

### 3.3. Perennials Dry Biomass

During the experiment, the variation in the perennials’ dry biomass was computed. In Table 6, the dry plant perennial biomass differences between the end and beginning of the experiment are reported.

**Figure 8:**
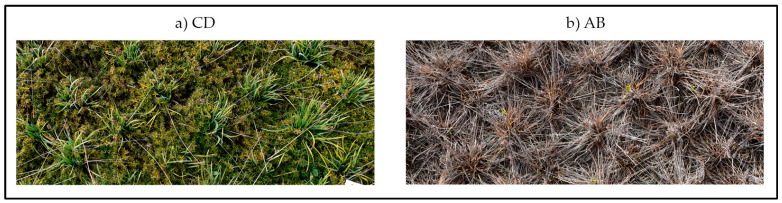
The best and the worst combinations, respectively, for producing perennial dry biomass during the three years at the end of the experiment (images taken after manual weeding performed on 12 November 2021). (**a**) *Tulbaghia violacea* Harv. + *Phlox subulata* L. “Trot Pink” (CD) with a dense and persistent vegetation. (**b**) *Hemerocallis* “Stella de Oro” + *Phedimus spurius* (M.Bieb.) “t Hart ‘John Creech” (AB) completely dried in autumn.

As highlighted in Table 6, the CD combination is able to produce a significantly higher biomass than the others, almost three-times more than the worst combination: AB. This result is in line with the findings previously reported. Indeed, the total amount of weeds at the end of the experiment for CD was the lowest and the ground cover during years 2 and 3 was the highest. Its better ability to produce an ornamental biomass than all of the other combinations tested was probably due to the rusticity of both perennial species and their growing habits (Figure 8a). Indeed, *Phlox subulata* L. “Trot Pink” with its ground cover habit produced extensive and dense vegetation. This species is also recognized in literature as having a good tolerance to drought stress, thanks to its metabolomic and physiological responses [49]. On the other hand, *Tulbaghia violacea* Harv., with its erect habit, did not compete with the other species (D) and produced a good and persistent vegetative mass. In contrast, the combination with *Hemerocallis* “Stella de Oro” + *Phedimus spurius* (M. Bieb.) “t Hart ‘John Creech” (AB) did not stand out either positively or negatively. Furthermore, it was the combination that, during the three years, produced the lowest ornamental biomass (Figure 8b). Effectively, we observed that, considering the species within the combination characterized by ground cover habit, *Phedimus spurius* (M. Bieb.) “t Hart ‘John Creech” was not able to produce vigorous vegetation in comparison with *Phlox subulata* L. “Trot Pink” of the CD combination. Furthermore, *Hemerocallis* “Stella de Oro” produced a non-persistent biomass during the entire vegetative season, differently by *Tulbaghia violacea* Harv. In fact, at the end of the experiment, after the last manual weeding in autumn, it was completely dry.

## 4. Conclusions

This research proposed an innovative methodological approach to evaluate the role of ornamental perennial herbaceous species in weed containment towards sustainable green urban area management. The first novelty was the growing performance evaluation of couples and combinations of couples of ornamental perennial species. Furthermore, the duration of the experiment allowed us to evaluate these performances over three years, allowing us to understand how the different combinations contributed as weed deterrents during different time periods. Indeed, some solutions appeared better than the others during the first year but were worse in the following years, suggesting the importance of mixing different plant habits, maximizing the performances and avoiding competition.

All the species tested showed a good adaptation to low-maintenance conditions, without irrigation and fertilization during the experiment. One of them (*Gaillardia* “Kobold”) better contributed during the first year to cover the soil rapidly. However, this species appeared interesting only for annual usage. The combination of *Tulbaghia violacea* Harv. and *Phlox subulata* L. “Trot Pink” showed the most constant ground cover during the years after the first and the best weed containment performance during the entire experiment. In contrast, the solutions with a higher number of species were the least efficient in avoiding the weeds, considering their total dry amount harvested during the three years. Indeed, the combination with the six species tested (AB + CD + EF) allowed for the growth of the highest total weed biomass.

The findings allowed us to identify the criteria to reduce green areas’ management costs, keep the ground covered, spare water, and avoid weeds. The findings of the research can be taken in consideration for future green designs of sustainable cities. In particular, some useful guidelines include the use of ornamental perennials to keep the ground covered and contain the weeds; the consideration of combinations of ornamental perennials characterized by different habits; and the preference of combinations with two or few species instead of solutions with many species existing together.

The research can be improved further in the future by evaluating the ornamental value of the different combinations of ornamental species with quantitative analyses and perceptive studies. The time and energy for weed removal operations can be explored in the future, in order to optimize green maintenance in an urban environment. Furthermore, other research can consider the use of spontaneous vegetation to strengthen biodiversity, avoiding exotic and invasive species. Indeed, identifying the solutions for a more sustainable green infrastructure design, increasing the biodiversity, and including high ornamental value is a potential and crucial challenge in the future. 

## Figures and Tables

**Table 1:** The life forms, habits, and blooming times of the six species selected for the research [38,39,40].

Species	Life Forms ^1^	Habits	Blooming Times
*Hemerocallis* “Stella De Oro”	Geo	Clump forming	Summer
*Phedimus spurius* (M.Bieb.) ‘t Hart ‘John Creech”	Ch	Mat forming	Summer
*Tulbaghia violacea* Harv.	Geo	Tufted	Summer/autumn
*Phlox subulata* L. “Trot Pink”	H	Mat forming	Spring
*Potentilla neumanniana* Rchb.	H	Clump forming	Spring/autumn
*Gaillardia* “Kobold”	H	Clump forming	Summer/autumn

^1^ Geo = geophytes (plants with belowground buds as storage organs, i.e., rhizomes or bulbs); Ch = chamaephytes (plants with buds 0.25 m above the soil surface); H = hemicryptophytes (plants with buds at or near the soil surface).

**Table 2:** The dates of the three manual weeding schedules per year.

Year	Season	Date
1	Spring	21 June 2019
	Summer	26 August 2019
	Autumn	5 November 2019
2	Spring	18 May 2020
	Summer	30 July 2020
	Autumn	22 December 2020
3	Spring	13 May 2021
	Summer	31 August 2021
	Autumn	12 November 2021

**Table 3:** Mean dry weed biomass per plot (g m^−2^) for years 1, 2, and 3 and total amount (g m^−2^) affected by seven perennial plant combinations: AB, CD, EF, AB + CD, CD + EF, AB + EF, and AB + CD + EF (*Hemerocallis* “Stella de Oro” (A), *Phedimus spurius* (M. Bieb.) “t Hart ‘John Creech” (B), *Tulbaghia violacea* Harv. (C), *Phlox subulata* L. “Trot Pink” (D), *Potentilla neumanniana* Rchb. (E), and *Gaillardia* “Kobold” (F)).

Combinations	Year 1	Year 2 ^1^	Year 3 ^1^	Total ^1^
AB	54.9 ± 42.2	4.9 ± 0.8 ^ab^	5.0 ± 0.4 ^ab^	778.5 ± 86.5 ^ab^
CD	26.6 ± 15.0	1.4 ± 0.5 ^b^	0.5 ± 0.3 ^b^	340.6 ± 17.6 ^b^
EF	9.2 ± 5.8	15.6 ± 6.1 ^ab^	14.7 ± 12.4 ^a^	474.1 ± 18.5 ^ab^
AB + CD	41.1 ± 27.3	6.0 ± 4.1 ^ab^	1.8 ± 0.3 ^ab^	586.2 ± 40.8 ^ab^
CD + EF	39.3 ± 33.5	19.9 ± 8.7 ^a^	15.9 ± 10.6 ^a^	902.0 ± 29.6 ^ab^
AB + EF	47.2 ± 39.7	29.5 ± 13.6 ^a^	8.6 ± 2.0 ^a^	1023.3 ± 57.1 ^ab^
AB + CD + EF	27.5 ± 22.9	52.0 ± 29.2 ^a^	13.4 ± 4.1 ^a^	1114.6 ± 127.0 ^a^
*p*	ns	***	***	*

^1^ Means sharing the same letter are not significantly different at *p* ≤ 0.05. The statistical relevance is provided (* *p* < 0.05; *** *p* < 0.001; ns = not significant).

**Table 4:** Two-way ANOVA was used to compare the mean percentages (%) of ground cover during years (1, 2, and 3), seasons (spring, summer, and autumn), and their interaction.

Year (A)	% ^1^
1	66.0 ± 1.6 ^a^
2	54.0 ± 5.2 ^b^
3	52.8 ± 10.3 ^c^
*p*	**
Season (B)	
Spring	76.5 ± 7.7 ^a^
Summer	54.2 ± 12.3 ^b^
Autumn	42.2 ± 8.4 ^c^
*p*	***
Interaction	*p*
A X B	***

^1^ Means sharing the same letter are not significantly different at *p* ≤ 0.05. The statistical relevance is provided (** *p* < 0.01; *** *p* < 0.001).

**Table 5:** The percentages (%) of ground cover by seven perennial plant combinations: AB, CD, EF, AB + CD, CD + EF, AB + EF, and AB + CD + EF (*Hemerocallis* “Stella de Oro” (A), *Phedimus spurius* (M.Bieb.) “t Hart ‘John Creech” (B), *Tulbaghia violacea* Harv. (C), *Phlox subulata* L. “Trot Pink” (D), *Potentilla neumanniana* Rchb. (E), and *Gaillardia* “Kobold” (F)) at each time point during the three-year-long experiment.

	Year 1			Year 2			Year 3		
Combinations	Spring ^1^	Summer	Autumn ^1^	Spring ^1^	Summer ^1^	Autumn ^1^	Spring ^1^	Summer ^1^	Autumn ^1^
AB	60.1 ± 3.9 ^bc^	55.4 ± 2.8	76.4 ± 2.5 ^ab^	96.0 ± 0.4 ^a^	37.4 ± 3.0 ^b^	2.1 ± 0.4 ^c^	92.0 ± 0.4 ^a^	8.4 ± 3.6 ^d^	0.5 ± 0.1 ^d^
CD	42.7 ± 3.3 ^d^	63.0 ± 2.8	84.5 ± 2.9 ^a^	94.3 ± 0.8 ^a^	70.5 ± 0.8 ^a^	24.5 ± 4.4 ^b^	84.6 ± 0.7 ^a^	65.0 ± 3.1 ^a^	43.4 ± 2.5 ^a^
EF	73.5 ± 1.1 ^a^	63.4 ± 4.7	66.3 ± 1.5 ^b^	51.5 ± 8.3 ^c^	65.5 ± 5.1 ^a^	42.8 ± 4.8 ^a^	69.7 ± 4.1 ^b^	72.2 ± 6.2 ^a^	39.4 ± 3.9 ^a^
AB + CD	52.6 ± 1.3 ^c^	66.0 ± 2.5	82.5 ± 2.6 ^a^	96.7 ± 0.3 ^a^	48.0 ± 1.4 ^b^	14.8 ± 1.5 ^bc^	94.6 ± 0.4 ^a^	26.7 ± 5.1 ^cd^	23.0 ± 3.4 ^bc^
CD + EF	66.1 ± 1.8 ^ab^	60.3 ± 5.2	73.5 ± 3.8 ^ab^	71.5 ± 7.5 ^b^	62.2 ± 2.7 ^a^	28.8 ± 4.7 ^ab^	65.7 ± 7.5 ^b^	69.8 ± 9.8 ^a^	37.8 ± 4.6 ^ab^
AB + EF	71.2 ± 1.1 ^a^	59.3 ± 5.7	69.8 ± 2.9 ^b^	87.8 ± 1.7 ^ab^	46.8 ± 2.4 ^b^	29.8 ± 4.2 ^ab^	93.6 ± 0.7 ^a^	37.0 ± 6.0 ^bc^	14.8 ± 3.5 ^c^
AB + CD + EF	65.8 ± 2.7 ^ab^	60.7 ± 3.7	72.9 ± 1.8 ^ab^	85.3 ± 6.9 ^ab^	49.0 ± 2.1 ^b^	28.5 ± 5.5 ^ab^	91.0 ± 1.5 ^a^	50.8 ± 2.4 ^ab^	29.1 ± 5.6 ^abc^
*p*	***	ns	***	***	***	***	***	***	***

^1^ Means sharing the same letter are not significantly different at *p* ≤ 0.05. The statistical relevance is provided (*** *p* < 0.001; ns = not significant).

**Table 6:** Dry plant perennial biomass (g m^−2^) differences between the end and beginning of the experiment as affected by seven perennial plant combinations: AB, CD, EF, AB + CD, CD + EF, AB + EF, and AB + CD + EF (*Hemerocallis* “Stella de Oro” (A), *Phedimus spurius* (M. Bieb.) “t Hart ‘John Creech” (B), *Tulbaghia violacea* Harv. (C), *Phlox subulata* L. “Trot Pink” (D), *Potentilla neumanniana* Rchb. (E), and *Gaillardia* “Kobold” (F)).

Combinations	Dry Perennial Biomass Variation ^1^
AB	1484.2 ± 0063.8 ^c^
CD	4316.8 ± 0526.9 ^a^
EF	2651.6 ± 0145.8 ^b^
AB + CD	2297.5 ± 0114.6 ^bc^
CD + EF	2899.7 ± 0111.2 ^b^
AB + EF	2257.9 ± 0128.6 ^bc^
AB + CD + EF	2150.3 ± 0087.4 ^bc^
*p*	***

^1^ Means sharing the same letter are not significantly different at *p* ≤ 0.05 (*** *p* < 0.001).

## Data Availability

Data available on request from the corresponding author.

## Supplementary Materials

The following supporting information can be downloaded at: https://www.mdpi.com/article/10.3390/plants12183293/s1.

## References

[1] Semeraro T., Scarano A., Buccolieri R., Santino A., Aarrevaara E. (2021). Planning of urban green spaces: An ecological perspective on human benefits. Land.

[2] Hamada S., Ohta T. (2010). Seasonal variations in the cooling effect of urban green areas on surrounding urban areas. Urban For. Urban Green..

[3] Aram F., García E.H., Solgi E., Mansournia S. (2019). Urban green space cooling effect in cities. Heliyon.

[4] Agenda 2030—United Nations Regional Information Centre. https://unric.org/it/agenda-2030/.

[5] Battisti L., Pomatto E., Larcher F. (2019). Assessment and mapping green areas ecosystem services and socio-demographic characteristics in Turin neighborhoods (Italy). Forests.

[6] Iojă C.I., Grădinaru S.R., Onose D.A., Vânău G.O., Tudor A.C. (2014). The potential of school green areas to improve urban green connectivity and multifunctionality. Urban For. Urban Green..

[7] Duan J., Wang Y., Fan C., Xia B., de Groot R. (2018). Perception of urban environmental risks and the effects of urban green infrastructures (UGIs) on human well-being in four public green spaces of Guangzhou, China. Environ. Manag..

[8] Larcher F., Pomatto E., Battisti L., Gullino P., Devecchi M. (2021). Perceptions of urban green areas during the social distancing period for COVID-19 containment in Italy. Horticulturae.

[9] Ugolini F., Massetti L., Calaza-Martínez P., Cariñanos P., Dobbs C., Ostoić S.K., Marin A.M., Pearlmutter D., Saaroni H., Šaulienė I. (2020). Effects of the COVID-19 pandemic on the use and perceptions of urban green space: An international exploratory study. Urban For. Urban Green..

[10] Sjöman H., Bellan P., Hitchmough J., Oprea A. (2015). Herbaceous plants for climate adaptation and intensely developed urban sites in Northern Europe: A case study from the Eastern Romanian Steppe. Ekologia.

[11] Sikorski P., Wińska-Krysiak M., Chormański J., Krauze K., Kubacka K., Sikorska D. (2018). Low-maintenance green tram tracks as a socially acceptable solution to greening a city. Urban For. Urban Green..

[12] Silvennoinen S., Taka M., Yli-Pelkonen V., Koivusalo H., Ollikainen M., Setälä H. (2017). Monetary value of urban green space as an ecosystem service provider: A case study of urban runoff management in Finland. Ecosyst. Serv..

[13] Nam J., Dempsey N. (2019). Understanding stakeholder perceptions of acceptability and feasibility of formal and informal planting in Sheffield’s district parks. Sustainability.

[14] Kühn N. (2006). Intentions for the unintentional: Spontaneous vegetation as the basis for innovative planting design in urban areas. J. Landsc. Archit..

[15] Capotorti G., Bonacquisti S., Abis L., Aloisi I., Attorre F., Bacaro G., Balletto G., Banfi E., Barni E., Bartoli F. (2020). More nature in the city. Plant Biosyst..

[16] Bonthoux S., Voisin L., Bouché-Pillon S., Chollet S. (2019). More than weeds: Spontaneous vegetation in streets as a neglected element of urban biodiversity. Landsc. Urban Plan..

[17] Vega K.A., Küffer C. (2021). Promoting wildflower biodiversity in dense and green cities: The important role of small vegetation patches. Urban For. Urban Green..

[18] Hoyle H., Jorgensen A., Warren P., Dunnett N., Evans K. (2017). “Not in their front yard” The opportunities and challenges of introducing perennial urban meadows: A local authority stakeholder perspective. Urban For. Urban Green..

[19] Hitchmough J. (2009). Diversification of grassland in urban greenspace with planted, nursery-grown forbs. J. Landsc. Archit..

[20] Caser M., Demasi S., Mozzanini E., Chiavazza P.M., Scariot V. (2022). Germination Performances of 14 Wildflowers Screened for Shaping Urban Landscapes in Mountain Areas. Sustainability.

[21] Mody K., Lerch D., Müller A.-K., Simons N.K., Blüthgen N., Harnisch M. (2020). Flower power in the city: Replacing roadside shrubs by wildflower meadows increases insect numbers and reduces maintenance costs. PLoS ONE.

[22] Bretzel F., Vannucchi F., Romano D., Malorgio F., Benvenuti S., Pezzarossa B. (2016). Wildflowers: From conserving biodiversity to urban greening—A review. Urban For. Urban Green..

[23] Jiang Y., Yuan T. (2017). Public perceptions and preferences for wildflower meadows in Beijing, China. Urban For. Urban Green..

[24] Southon G.E., Jorgensen A., Dunnett N., Hoyle H., Evans K.L. (2018). Perceived species-richness in urban green spaces: Cues, accuracy and well-being impacts. Landsc. Urban Plan..

[25] Southon G.E., Jorgensen A., Dunnett N., Hoyle H., Evans K.L. (2017). Biodiverse perennial meadows have aesthetic value and increase residents’ perceptions of site quality in urban green-space. Landsc. Urban Plan..

[26] Lindemann-Matthies P., Brieger H. (2016). Does urban gardening increase aesthetic quality of urban areas? A case study from Germany. Urban For. Urban Green..

[27] Steven Colombo A., Berto R., Ferrario P., Toccolini A., Larcher F. (2020). Wildflowers in urban design: An exploratory research of preference in Italian adults. Vis. Sustain..

[28] Sikora A., Michołap P., Sikora M. (2020). What kind of flowering plants are attractive for bumblebees in urban green areas?. Urban For. Urban Green..

[29] Hitchmough J., Wagner M., Ahmad H. (2017). Extended flowering and high weed resistance within two layer designed perennial “prairie-meadow” vegetation. Urban For. Urban Green..

[30] Krzyżak J., Rusinowski S., Sitko K., Szada-Borzyszkowska A., Borgulat J., Stec R., Hanslin H.M., Pogrzeba M. (2023). The Effect of Combined Drought and Temperature Stress on the Physiological Status of Calcareous Grassland Species as Potential Candidates for Urban Green Infrastructure. Plants.

[31] Marble S.C., Koeser A.K., Hasing G., McClean D., Chandler A. (2017). Efficacy and estimated annual cost of common weed control methods in landscape planting beds. HortTechnology.

[32] Marble S.C., Koeser A.K., Hasing G. (2015). A review of weed control practices in landscape planting beds: Part I–nonchemical weed control methods. HortScience.

[33] Marble S.C., Koeser A.K., Hasing G. (2015). A review of weed control practices in landscape planting beds: Part II—Chemical weed control methods. HortScience.

[34] Duarte D., Galhano C., Dias M.C., Castro P., Lorenzo P. (2023). Invasive plants and agri-food waste extracts as sustainable alternatives for the pre-emergence urban weed control in Portugal Central Region. Int. J. Sustain. Dev. World Ecol..

[35] Caser M., Demasi S., Caldera F., Dhakar N.K., Trotta F., Scariot V. (2020). Activity of *Ailanthus altissima* (Mill.) swingle extract as a potential bioherbicide for sustainable weed management in horticulture. Agronomy.

[36] Foo C., Harrington K., MacKay M. (2011). Weed suppression by twelve ornamental ground cover species. N. Z. Plant Prot..

[37] Agenzia Regionale per la Protezione Ambientale-Arpa Piemonte Meteorological Database. https://www.arpa.piemonte.it/rischinaturali/accesso-ai-dati/annali_meteoidrologici/annali-meteo-idro/banca-dati-meteorologica.html.

[38] Acta Plantarum. httpss://www.actaplantarum.org/flora/flora.php.

[39] Pladias. https://pladias.cz/en/taxon/data/Phlox%20subulata.

[40] Royal Horticultural Society 2023—Find a Plant. https://www.rhs.org.uk/plants/search-form.

[41] Xiong Y., West C., Brown C., Green P. (2019). Digital image analysis of old world bluestem cover to estimate canopy development. Agron. J..

[42] Patrignani A., Ochsner T.E. (2015). Canopeo: A powerful new tool for measuring fractional green canopy cover. Agron. J..

[43] Eom S.H., Senesac A.F., Tsontakis-Bradley I., Weston L.A. (2005). Evaluation of herbaceous perennials as weed suppressive groundcovers for use along roadsides or in landscapes. J. Environ. Hortic..

[44] Toscano S., Ferrante A., Romano D. (2019). Response of Mediterranean ornamental plants to drought stress. Horticulturae.

[45] Süle G., Miholcsa Z., Molnár C., Kovács-Hostyánszki A., Fenesi A., Bauer N., Szigeti V. (2023). Escape from the garden: Spreading, effects and traits of a new risky invasive ornamental plant (*Gaillardia aristata* Pursh). NeoBiota.

[46] Zollinger N., Kjelgren R., Cerny-Koenig T., Kopp K., Koenig R. (2006). Drought responses of six ornamental herbaceous perennials. Sci. Hortic..

[47] Martinetti L., Tosca A., Spoleto P., Valagussa M., Gatt A. Evaluation of water stress tolerance of some species suitable for extensive green roofs. Proceedings of the International Symposium on Greener Cities for More Efficient Ecosystem Services in a Climate Changing World 1215.

[48] Tosca A., Martinetti L., Spoleto P., Valagussa M., Gatt A. Monitoring of weeds in an extensive green roof. Proceedings of the International Symposium on Greener Cities for More Efficient Ecosystem Services in a Climate Changing World 1215.

[49] Xiong Y., Qu Y., Han H., Chen F., Li L., Tang H., Che D., Zhang X. (2021). Unraveling physiological and metabolomic responses involved in *Phlox subulata* L. tolerance to drought stress. Plant Mol. Biol. Report..

