# Supplementary material for: Evaluation of Different Combinations of Ornamental Perennials for Sustainable Management in Urban Greening
Source: Plants (Basel). 2023 Sep 18;12(18):3293. doi: 10.3390/plants12183293 (PMC10536764; doi:10.3390/plants12183293)
Supplement: Supplementary file 1 [file plants-12-03293-s001.zip › plants-2558426-supplementary.pdf]

plants MDPI

Evaluation of Different Combinations of Ornamental Perennials for Sustainable Management in Urban Greening

Enrico Pomatto [†], Federica Larcher [†,*], Matteo Caser, Walter Gaino and Marco Devecchi

Department of Agricultural, Forest and Food Sciences, University of Turin, Largo Paolo Braccini 2, 10095, Grugliasco, Italy; enrico.pomatto@unito.it (E.P.); federica.larcher@unito.it (F.L.); matteo.caser@unito.it (M.C.); walter.gaino@unito.it (W.G.), marco.devecchi@unito.it (M.D.)
† These authors contributed equally to this work
* Correspondence: federica.larcher@unito.it

Supplemetary materials

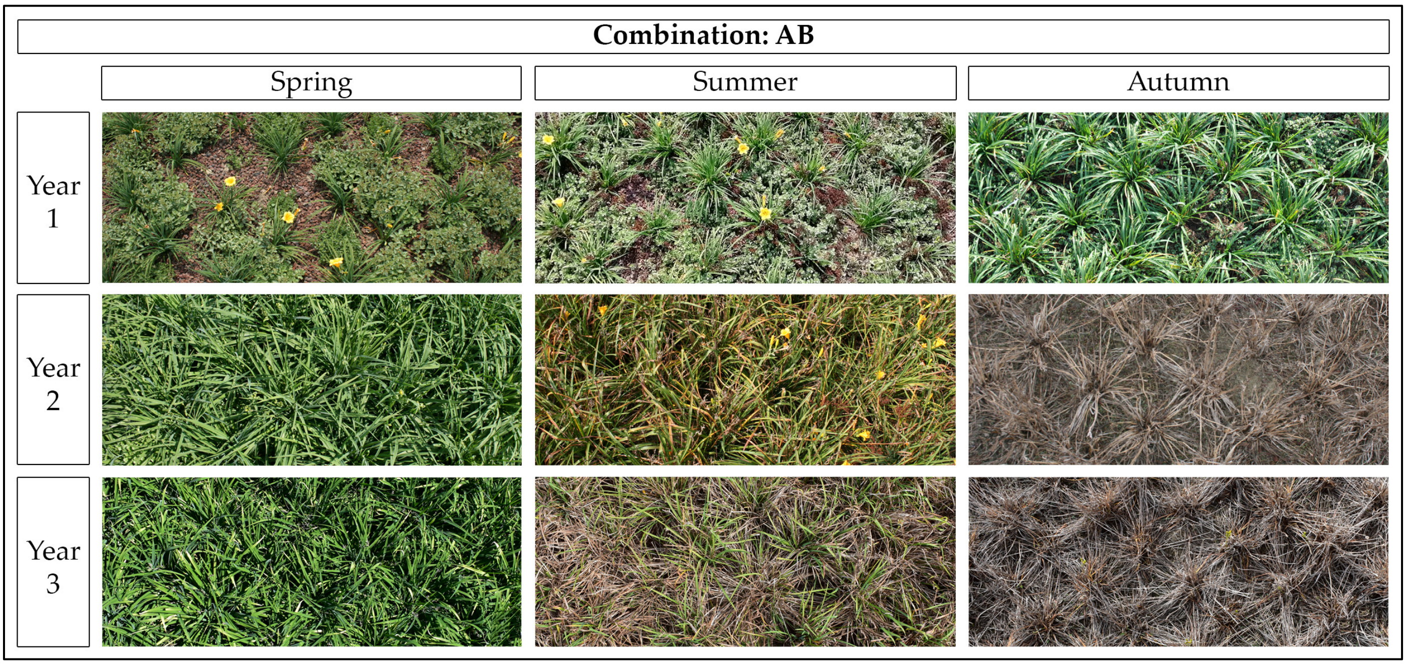

Figure S1. The evolution over time of the AB combination (*Hemerocallis* 'Stella de Oro' (A), *Phedimus spurius* (M.Bieb.) 't Hart 'John Creech' (B)).

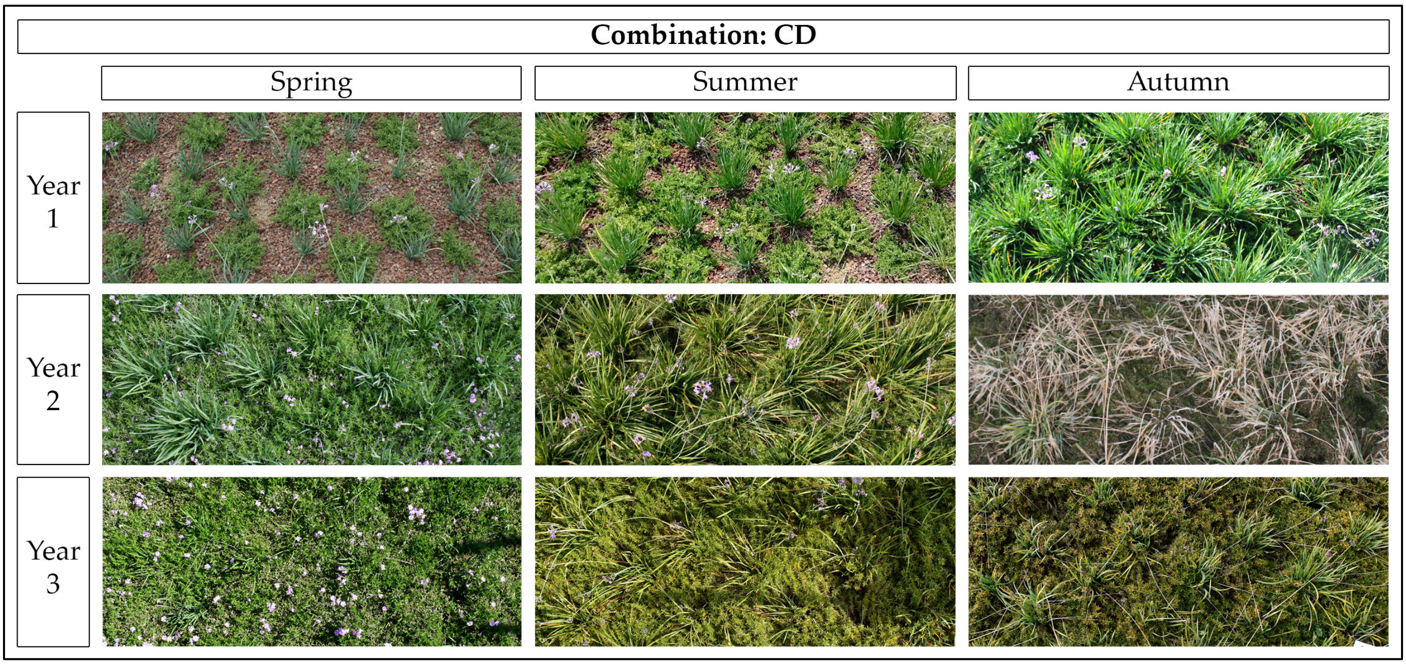

Figure S2. The evolution over time of the CD combination (*Tulbaghia violacea* Harv. (C), *Phlox subulata* L. 'Trot Pink' (D)).

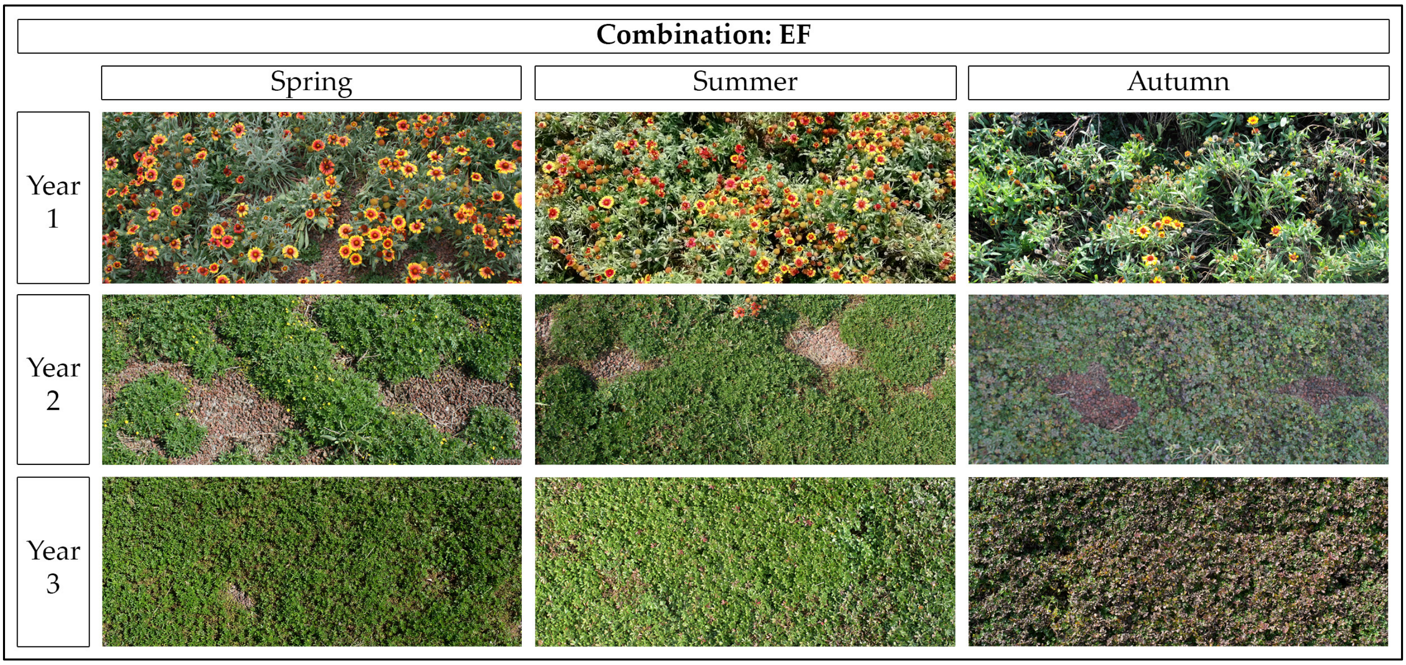

Figure S3. The evolution over time of the EF combination (*Potentilla neumanniana* Rchb. (E) and *Gaillardia* 'Kobold' (F)).

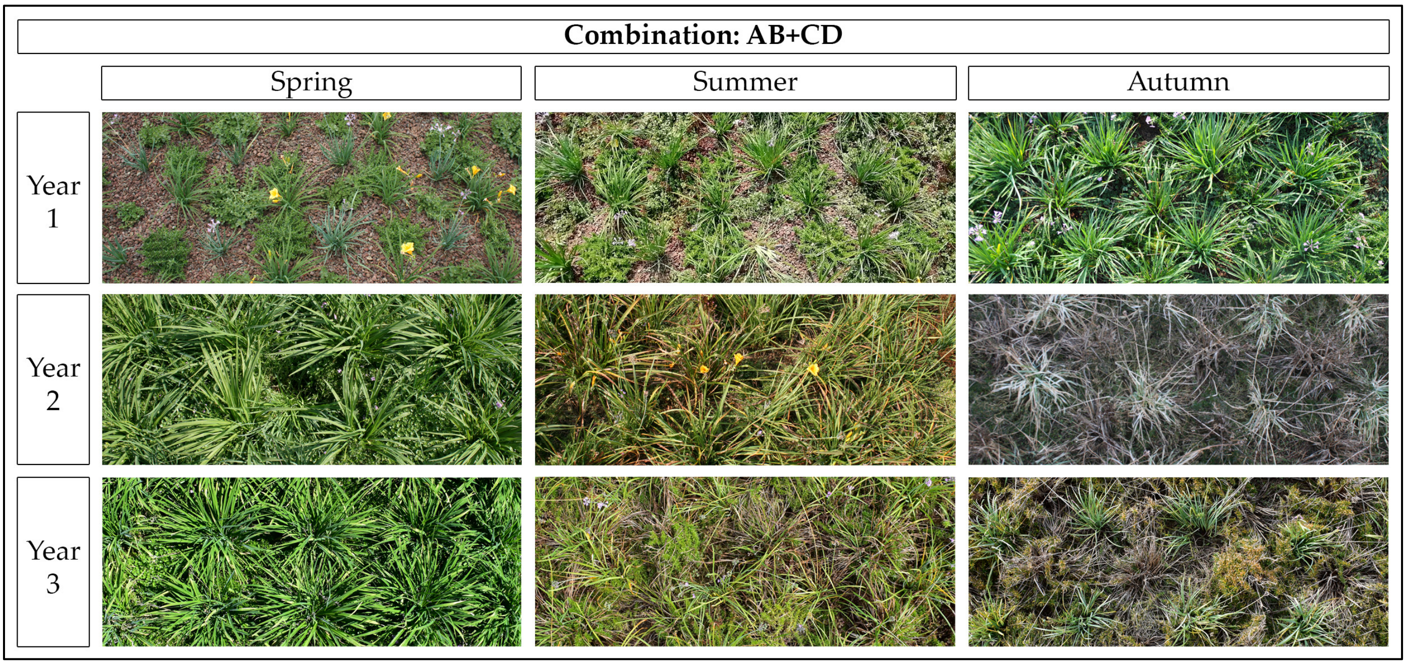

Figure S4. The evolution over time of the AB+CD combination (*Hemerocallis* 'Stella de Oro' (A), *Phedimus spurius* (M.Bieb.) 't Hart 'John Creech' (B), *Tulbaghia violacea* Harv. (C), *Phlox subulata* L. 'Trot Pink' (D)).

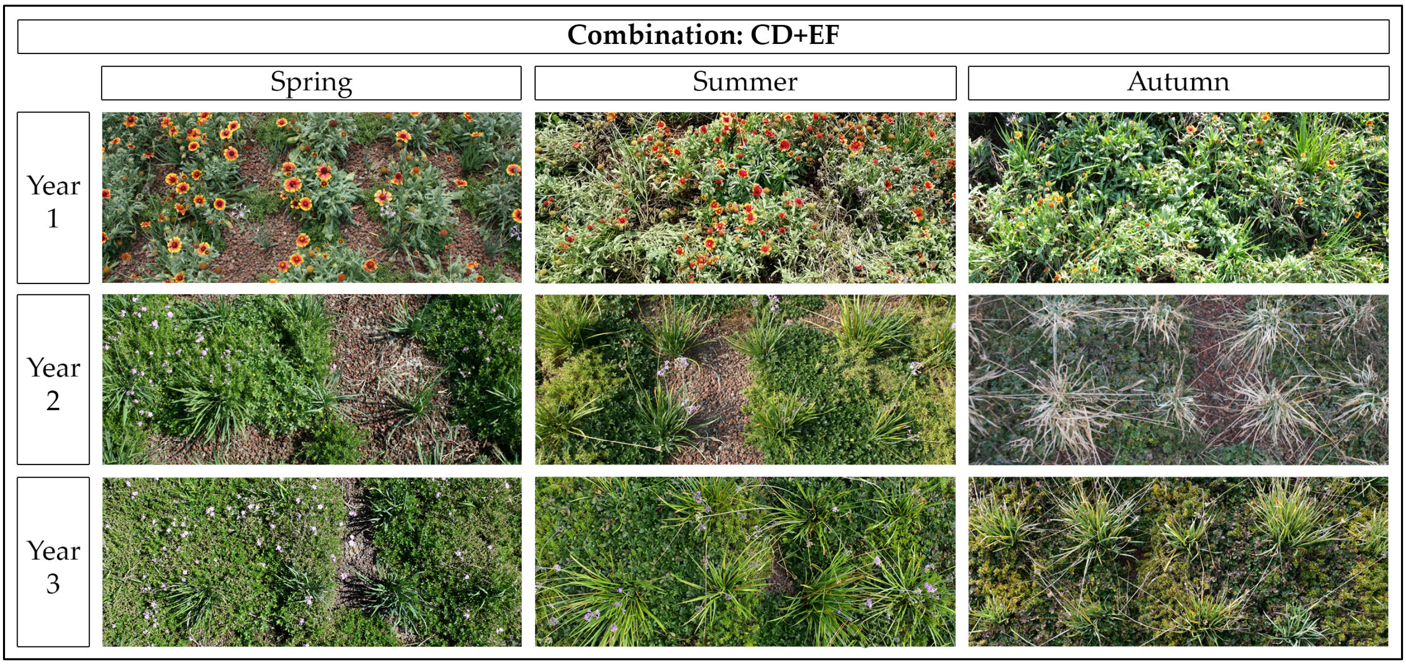

Figure S5. The evolution over time of the CD+EF combination (*Tulbaghia violacea* Harv. (C), *Phlox subulata* L. 'Trot Pink' (D), *Potentilla neumanniana* Rchb. (E) and *Gaillardia* 'Kobold' (F)).

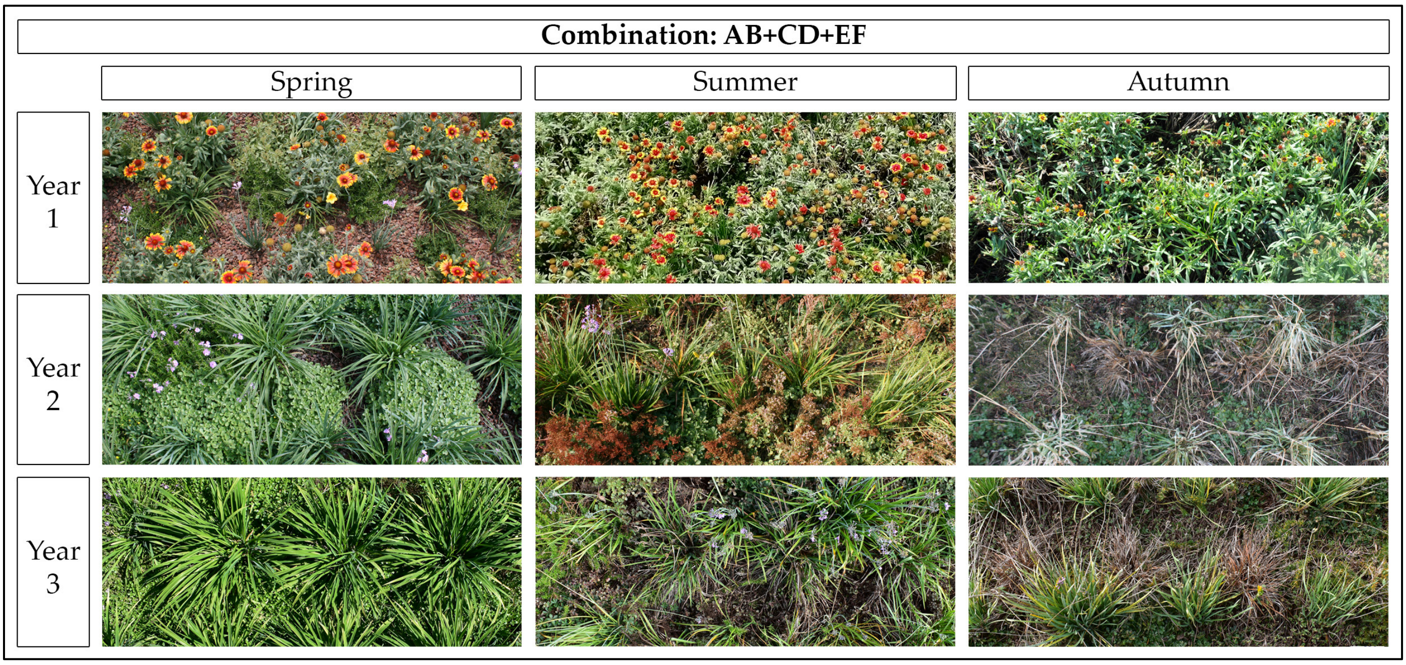

Figure S6. The evolution over time of the AB+CD+EF combination (*Hemerocallis* 'Stella de Oro' (A), *Phedimus spurius* (M.Bieb.) 't Hart 'John Creech' (B), *Tulbaghia violacea* Harv. (C), *Phlox subulata* L. 'Trot Pink' (D), *Potentilla neumanniana* Rchb. (E) and *Gaillardia* 'Kobold' (F)).
